# Rupture of Gallbladder

**Published:** 1905-04

**Authors:** Benjamin Merrill Ricketts

**Affiliations:** Cincinnati, Ohio.


### ABSTRACTS.

### Rupture of Gallbladder.

SPONTANEOUS AND TRAUMATIC, OPERATIVE AND NONOPERATIVE—A HISTORICAL REVIEW OF 203 CASES.

BY BENJAMIN MERRILL RICKETTS, PH.D., M. D., Cincinnati, Ohio.

AUTHOR'S ABSTRACT.

[*Saint Louis Medical Review*, February 18, 1905.]

THE author in the introductory paragraphs states that the object of his paper is to present as briefly as possible, the more important points of all available cases of spontaneous and traumatic rupture of the gallbladder, which have been reported to date in the hope, that the comparison of the results of operative and nonoperative methods of treatment thus afforded may be of use both to the physician and surgeon.

To facilitate this comparison he has divided the 203 cases mentioned into two classes, the spontaneous and the traumatic rupture; and each of these classes in turn into four divisions, each of which he treats in a separate chapter.

In chapter I, under the heading of spontaneous rupture he mentions 37 cases of spontaneous rupture of the gallbladder, which have been operated on successfully, and shows that 80 per cent. of these have been females; that one or more concretions have been found in 72 per cent of them; that 80 per cent. of these successful operations have been cholecystotomies.

In chapter II he mentions 27 cases which have been operated on successfully and shows that they were equally distributed between males and females; that concretions have been found in about 60 per cent. of them; and that 80 per cent. of these unsuccessful operations have been abdominal sections.

In chapter III he mentions 6 cases which have recovered without operation, and in chapter IV, 89 cases which have died without operation, showing that sex has been equally involved in these cases, and that concretions have been found in about 60 per cent. of them.

A final comparison shows the recovery of 58 per cent. of those operated on as against the recovery of only 6 per cent. of those not operated on. In chapter I, under the heading of traumatic rupture, he mentions 23 cases which have been operated on successfully, and in chapter II, 3 cases which have been operated on unsuccessfully, death having been due to shock, infection and hemorrhage.

In chapter III, he records 14 cases in which death resulted without operation, showing peritonitis to have been the ruling cause of death, and in chapter IV, 4 cases which have recovered without operation, recovery having probably been due to drainage. Comparison of all traumatic ruptures shows the recovery of 88 per cent. of those operated on as against the recovery of only 22 per cent. of those not operated on.

